# Resveratrol and Coumarin: Novel Agricultural Antibacterial Agent against *Ralstonia solanacearum* In Vitro and In Vivo

**DOI:** 10.3390/molecules21111501

**Published:** 2016-11-09

**Authors:** Juanni Chen, Yanmei Yu, Shili Li, Wei Ding

**Affiliations:** Laboratory of Natural Product Pesticide, College of Plant Protection, Southwest University, Chongqing 400715, China; chenhuanni521@126.com(J.C.); trcmei@126.com (Y.Y.); lsl203lst@163.com (S.L.)

**Keywords:** resveratrol, Coumarin, *R. solanacearum*, antibacterial activity, bacterial wilt

## Abstract

Bacterial wilt is a destructive disease caused by the phytopathogen *Ralstonia solanacearum* (*R. solanacearum*), which is widely found in various tobacco-growing areas all over the world. Botanical bactericidal substances have gradually emerged as a hot topic in modern pesticide research. In this study, the antibacterial activities of two phytochemicals (resveratrol and coumarin) against *R. solanacearum* and their in vivo and in vitro efficacy for controlling tobacco bacterial wilt were evaluated. We rule out significant biological effects of both phytochemicals using transmission electron microscope (TEM) and fluorescence microscope, which suppressed the growth of *R. solanacearum*. Furthermore, we demonstrated that the toxicity mechanisms mainly involved damaging bacterial cell membrane and preventing swarming motility and biofilm formation. A further pot experiment demonstrated that coumarin and resveratrol significantly inhibited early adhesion and colonization of *R. solanacearum* in tobacco plants and the corresponding control efficacies were 68% and 85% after incubation for 13 days, respectively. The findings of this study suggest that both resveratrol and coumarin have potential as non-toxic antimicrobial strategies for controlling tobacco bacterial wilt disease.

## 1. Introduction

*Ralstonia solanacearum,* belonging to the β-proteobacteria, is a soil-borne phytopathogenic bacterium that can cause bacterial wilt disease with destructive damage to a large number of economic crops, as well as some ornamentals. With the characteristic of genetic polymorphism in *R. solanacearum*, including worldwide geographic distribution and extraordinarily broad host range, bacterial wilt is one of the most devastating diseases in agriculture [1,2]. Usually, *R. solanacearum* is mainly distributed in the tropics, subtropics, and partly in the temperate belt, and was first described in tobacco in 1908. As a result of long-term co-evolution with the host and environment, *R. solanacearum* exhibits a high degree of complexity in its geographic distribution, host range, and pathogenetic mechanisms, making it difficult to prevent from infecting the host plants.

Control measures against *R. solanacearum* have long relied on traditional chemical pesticides (e.g., zinc thiazole, bismerthiazol, and saisentong) and antibiotics in many countries. However, limited efficacy and microbial resistance strains induced by uncontrolled application of chemical pesticides are a growing problem. Moreover, the use of these methods for field trial is largely limited due to their environmental pollution and potential health risks to human health or non-target organisms [3]. In recent years, increasing research has focused on agricultural and biological control for bacterial wilt disease management, such as applying soil additives [4], the adoption of resistant varieties, sterile grafts, and crop rotation [5,6,7]. However, plant bacterial wilt disease is still a challenging subject in agricultural crop protection. Therefore, there is an immediate need to search for new alternative antibacterial agents that can be used for managing *R. solanacearum.*

Plants are rich in natural antimicrobial substances, named botanical antimicrobial substances. Botanical pesticides and their derivatives have recently attracted extensive attention from chemists and biologists because of their low phytotoxicity, environment friendly effects, rapid environmental degradation, abundant resources, low cost and because they are renewable [8,9]. Wisely, combined with biotechnology advancement, plant-derived natural products have become a major trend in the development of modern pesticides. Up to now, it has been demonstrated that botanical antimicrobial substances from various plants (Gramineae, Labiatae, Apocynaceae, Rutaceae, Liliaceae and Magnoliaceae) [10,11,12,13], including secondary metabolites, have highly efficient sources of antimicrobial activity against agricultural phytopathogens [14,15,16,17] (Table 1). A great number of plant essential oils have been tested for their antimicrobial activities. A number of natural pesticides such as methyl gallate [18], Lansiumamide B [19], and essential oils [10,20] have been used to inhibit the growth of *R. solanacearum* and control the phytopathogenic diseases caused by them. In addition, the in vitro and in vivo activities of protocatechualdehyde, a natural polyphenol compound isolated from the roots of traditional Chinese medicine radix *Salviae Miltiorrhizae*, were evaluated against phytopathogen *R. solanacearum* [21].

Coumarin is a benzopyrone that occurs naturally in a wide variety of plants (including tonka bean, sweet clover, cinnamon oil and lavender) and essential oils, and they has been reported to exhibit high antifungal activity [22]. Resveratrol is a natural phytoalexin that is the biologically active ingredient of spermatophyte including red wine, grapes, peanuts, and strawberries, in response to environmental stress, and is commonly used to preserve food and beverages. Both resveratrol and coumarin have multiple biological activities and pharmacological effects that can inhibit the growth of human pathogenic bacteria and fungi [23,24,25], which, more significantly, have been extensively used medicinally for anticancer, anticoagulant, and antimicrobial purposes [23,25,26,27,28]. However, to our knowledge, there have been few reports regarding the effects of resveratrol and coumarin on plant pathogen *R. solanacearum*.

From another point of view, according to existing research, phytochemicals prevent or control plant pathogens mainly via two mechanisms. It was demonstrated that phytochemicals can directly induce damage to the cell wall and cytomembrane of pathogen, thereby altering cell morphology and permeability [29]. On the other hand, phytochemicals can efficiently manage diseases by increasing host resistance. Their application induces the expression of some plant proteins or polysaccharides such as clerodendrum aculeatum-systemic resistance inducing protein (CA-SRIP), glycoproteins and SL-polysaccharides, which can induce and activate host immune mechanisms to achieve the control effect [30]. In our previous work, we demonstrated antibacterial activities of a series of coumarins against *R. solanacearum* with different substitution patterns, and the results found that hydroxylation at the C-6, C-7 or C-8 position significantly enhanced the antibacterial activity of coumarins [31]. However, the research was limited to laboratory test.

Therefore, based on the concept of healthy cultivation and safe production in modern agriculture, the current study was performed to examine the antibacterial activity of two phytochemicals (resveratrol and coumarin) against *R. solanacearum* in vivo and in vitro, and to evaluate the possible antibacterial mechanism of action of these compounds by inhibitory effect assay, fluorescence microscope and transmission electron microscopy observations. Further, their disease control efficacy is also tested. This study provides new ideas for investigating the inhibitory mechanism of phytochemicals against *R. solanacearum* and studying the prevention and control of bacterial wilt of tobacco. The results lay a solid theoretical and practical foundation for the research and development of new biological pesticides.

## 2. Results

### 2.1. Determination of MIC and MBC

**Figure 1:**
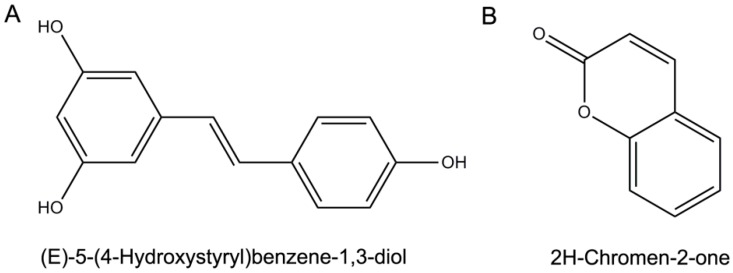
Molecular structural formula and chemical names of: resveratrol (**A**); and coumarin (**B**).

**Figure 2:**
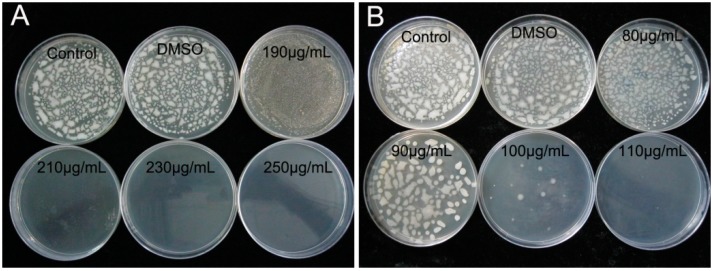
The colony growth of *R. solanacearum* treated with: (**A**) resveratrol at concentrations ranging from 190 to 250 μg/mL; and (**B**) coumarin at concentrations ranging from 80 to 110 μg/mL.

Resveratrol and coumarin are naturally obtained from plant and their molecular structure formulas are shown in Figure 1. As shown in Table 2, the minimum inhibitory concentrations (MICs) of resveratrol and coumarin in liquid medium were determined as 2 and 8 μg/mL, respectively, using a typical microdilution method (ISO 10932|IDF 223:2010). Phytochemicals of less than the MICs had no inhibitory effect on *R. solanacearum* in the liquid medium (Table 2). The MBCs of the two phytochemicals were measured by plate counting method, and the number of colony grown on phytochemical-containing plates was recorded after 96 h of incubation. The MBCs of resveratrol and coumarin against *R. solanacearum* were substantially different, 230 and 110 μg/mL, respectively (Figure 2).

### 2.2. Inhibition of R. solanacearum Growth

**Figure 3:**
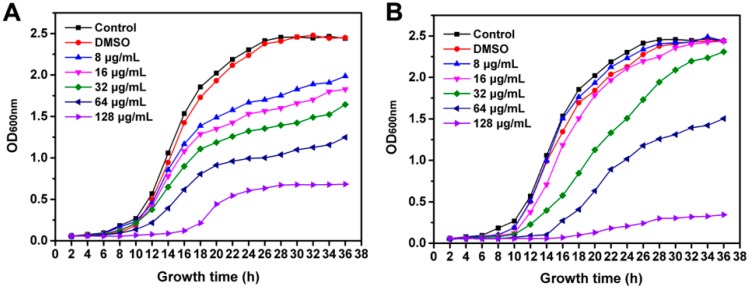
The bacterial growth curve of *R. solanacearum* in the presence of resveratrol (**A**) and coumarin (**B**) at concentrations ranging from 8 to 128 μg/mL. DMSO was indicated as a negative control and Control was used as bacterial cells treatmented with sterile water.

Usually, the antimicrobial activity of compound can be observed by measuring the bacterial growth curve. When *R. solanacearum* is grown under normal culture conditions, the growth and reproduction period is divided into the lag phase (0–8 h) with less growth, the logarithmic growth phase (8–22 h) with massive growth, and the stationary phase (after 24 h) with stable growth and a gradually decreasing reproduction rate. We plotted the growth curves of *R. solanacearum* in culture medium in the presence of different concentration of phytochemicals. The same range of concentration was selected between the MIC and MBC of resveratrol and coumarin. As observed in Figure 3, the results showed that both resveratrol and coumarin caused a growth delay of *R. solanacearum*, depending on the incubation concentration.

It can be seen from the results that, in the presence of resveratrol, *R. solanacearum* failed to enter the normal growth cycle. With increasing phytochemical concentration, the logarithmic and stationary phases of *R. solanacearum* were delayed. It is important to note that the bacteria growth rate significantly decreased with increasing concentration. When the bacteria were treated with 8 μg/mL of resveratrol, there was no major difference versus the control in the beginning; however, bacterial growth became slower after 18 h of incubation. With 128 μg/mL resveratrol, *R. solanacearum* entered the growth phase after 18 h of incubation and reached the stationary phase after 24 h of incubation; thereafter, no major changes were observed in the bacterial count, displaying significantly inhibitory effects on the bacterial growth (Figure 3A). Similarly, Figure 2B shows that the growth of *R. solanacearum* cells treated with various concentration of coumarin. Compared with the control, low concentrations (8 and 16 μg/mL) of coumarin had no obvious effects on bacterial growth; however, 32 and 64 μg/mL treatments significantly inhibited the logarithmic and stationary phases of *R. solanacearum*. Most importantly, bacterial growth was almost completely inhibited by coumarin under 128 μg/mL treatment (Figure 3B).

### 2.3. Bacterial Cell Morphology Observations

**Figure 4:**
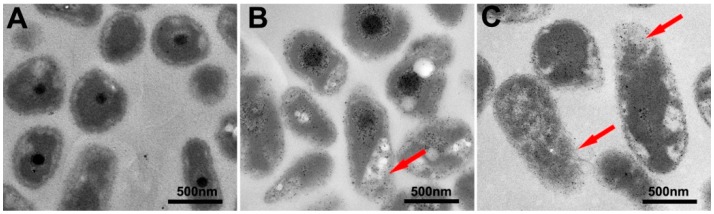
TEM images of *R. solanacearum* cells after incubation with: sterile water (**A**); resveratrol (**B**); and coumarin (**C**) for 2 h with final concentration 128 μg/mL.

We used TEM to observe cell morphology changes in *R. solanacearum* when they interacted with the phytochemicals. *R. solanacearum* cells were treated with resveratrol and coumarin at 128 μg/mL. In the control groups without phytochemical treatment, the cells were intact and uniform in size, with a clear boundary and a smooth surface (Figure 4A). When bacteria were treated with resveratrol, the cell morphology appeared incomplete and turned flat in a dissolved shape, with an unclear boundary (Figure 4B). After treatment with high concentration (128 μg/mL) of coumarin, more severe damage on cell membrane was found, some distorted projections emerged on the cell membrane, and the cell morphology was imperfect, flattened, and partially ruptured (Figure 4C).

### 2.4. Suppress the Swarming Motility and Biofilm Formation of R. solanacearum

**Figure 5:**
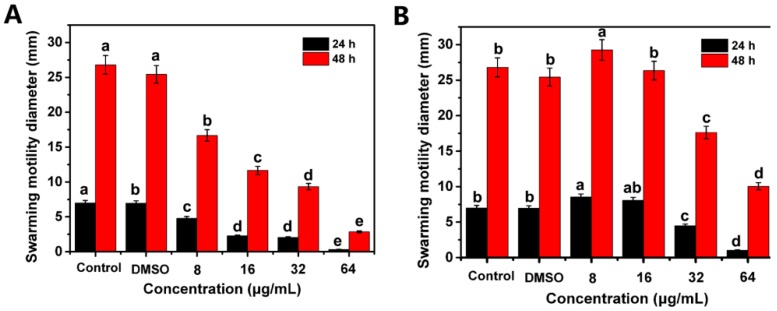
Effects of resveratrol (**A**) and coumarin (**B**) at different concentration on swarming motility of *R*. *solanacearum.* Error bars represent the standard deviation (*n* = 3). Values presented are means and standard errors from four independent experiments. Different letters indicate that the means are significantly different at *p* = 0.05.

Previous studies, to the best of our knowledge, have reported that flagella-driven swarming motility of *R. solanacearum* was extremely important to its virulence and contributed significantly to its invasion of host plants. We used the SMM semi-solid medium to evaluate the effects of resveratrol and coumarin on the swarming motility of *R. solanacearum*. The results displayed that both resveratrol and coumarin restrained the motility diameter of *R. solanacearum*, revealing significant differences compared with the control group. After incubation for 24 and 48 h, the motility diameter of *R. solanacearum* were strongly suppressed by resveratrol at different concentrations ranging from 8 to 64 μg/mL in comparison to DMSO and control treatment. The inhibitory effect increased with the rise in resveratrol concentrations. We analyzed the statistical significance using Duncan’s multiple comparison test (*p* ≤ 0.05) and found significant differences between various concentrations of resveratrol treatment and the control (Figure 5A). Coumarin had slight or no effect on the swarming motility of *R. solanacearum* at low concentrations (8 and 16 μg/mL). However, at 32 and 64 μg/mL coumarin, bacterial motility was significantly inhibited after both 24 and 48 h of incubation (Figure 5B). Taken together, these results suggest that resveratrol and coumarin inhibited the swarming motility of *R. solanacearum* in a concentration-dependent manner.

**Figure 6:**
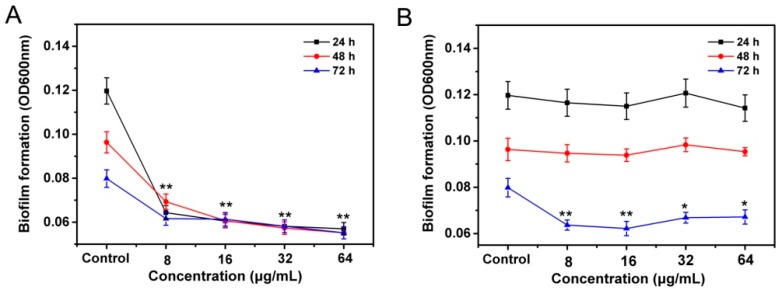
Effects of resveratrol (**A**) and coumarin (**B**) at different concentration on biofilm formation of *R. solanacearum*. Error bars represent the standard deviation (*n* = 3). * and ** indicate *p* < 0.05 and *p* < 0.01, respectively.

Further, we used a microplate reader to measure the optical density value at 490 nm (OD_490_) of bacterial biofilms to assess the effects of resveratrol and coumarin on the biofilm-forming ability of *R. solanacearum*. Bacterial biofilms are a form of bacterial growth on solid surfaces that corresponds to the migratory form of the cells [32]. For most plant pathogens, biofilms constitute a major part of several plant–bacterium interactions, and are involved in locating and infecting host plant roots [33,34]. The current results showed that under the experimental conditions, *R. solanacearum* exhibited the highest biofilm-forming ability at 24 h of incubation, with an OD_490_ of 0.120. Biofilm-forming ability gradually decreased with increasing time of incubation, with OD_490_ = 0.096 and 0.080 after 48 and 72 h, respectively. It is worth noting that treatment concentration plays an important effector role in this interaction process. Differently, the biofilm formation of *R. solanacearum* was sharply repressed in the presence of resveratrol, even at the low concentration of 8 μg/mL, with the minimum OD_490_ value of 0.057 after incubation for 24 h (Figure 6A).However, Coumarin had nearly no inhibitory effects on biofilm formation at 24 or 48 h of incubation (Figure 6B), while showed a slight inhibitory effect on biofilm formation of *R. solanacearum* at 72 h.

**Figure 7:**
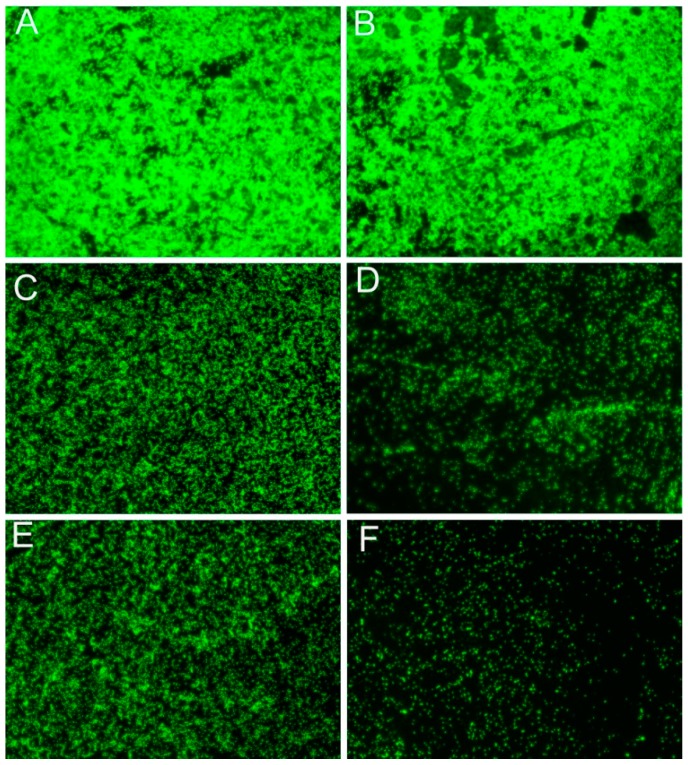
Fluorescence microscope images of biofilm formation of *R. solanacearum* cells after treatment with: (**A**) sterile water; (**B**) DMSO; (**C**,**D**) coumarin at final concentration of 64 μg/mL and 128 μg/mL respectively; and (**E**,**F**) carvacrol at final concentration of 64 μg/mL and 128 μg/mL, respectively. *R. solanacearum* were incubated with different treatment for 12 h at 30 °C without shaking, and exopolysaccharide matrixes were stained with FITC-ConA. Each treatment was repeated in triplicate.

The biofilm inhibitory effects activity of phytochemical against phytopathogens was further substantiated using fluorescent imaging. Fluorescein isothio-cyanate-conjugated concanavalin A (FITC-ConA) dye was used to examine the effects of the two phytochemicals on biofilm formation by *R. solanacearum*. FITC-ConA can stain extracellular polysaccharides, which is the major component of bacterial biofilms, and emits green fluorescence [35]. The results demonstrated that in the absence of both phytochemical (control group), *R. solanacearum* biofilm adhered to the carrier in large pieces and formed aggregates of flocculent green fluorescent material (Figure 7A,B). With coumarin (Figure 7C,D) and resveratrol (Figure 7E,F) treatment at 64 μg/mL, only a few bacterial cells adhered to the carrier in a dispersed pattern; the green fluorescent material was markedly reduced and thinned compared with the control. At a higher phytochemical concentration (128 μg/mL), it seems that the number of adherent bacterial cells was reduced even further, while green fluorescent material clustered and overlapped, with enhanced fluorescence. These results further demonstrated that both resveratrol and coumarin inhibited the formation of bacterial biofilms, in agreement with the OD_490_ measurements.

### 2.5. Effects of Phytochemicals on Bacterial Adhesion and Colonization

Typical infection of a host plant by *R. solanacearum* involves three stages: root surface colonization, root cortex infection, and xylem parenchyma infection and invasion [36]. Here, we evaluated the effects of resveratrol and coumarin on bacterial colonization on the tobacco plant. Tobacco plants were soil-soak inoculated with concentration of 10 mL, 1 × 10^8^ cfu/mL of *R. solanacearum* and incubated at 28 ± 1 °C after irrigating roots with coumarin and resveratrol. Following non-injured root inoculation, resveratrol and coumarin displayed inhibitory effects on the ability of *R. solanacearum* to adhere to and colonize tobacco roots (Table 3).

**Figure 8:**
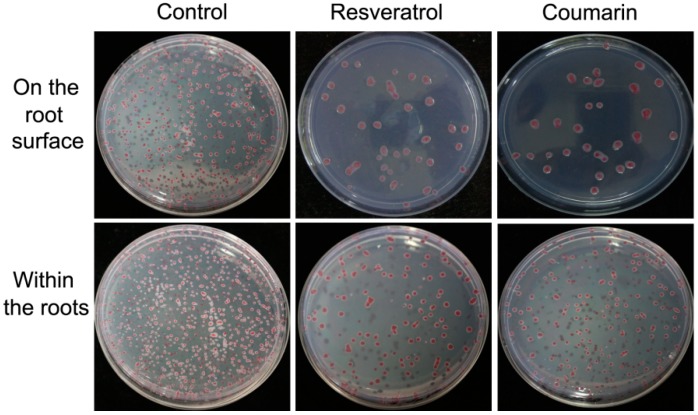
Representative SMSA-agar plate images of *R. solanacearum* colony in root exterior and interior after treatment with 128 μg/mL of coumarin and resveratrol on Day 5 after inoculation. After treatment with both phytochemical for five days, 10^−3^ to 10^−5^ bacterial suspensions were used for the test.

In the control, the bacterial count on the tobacco root surface increased with increasing inoculation time. However, no bacteria were detected in the root interior on Day 1 after inoculation, and the bacterial count increased on Day 3 after inoculation. In the coumarin and resveratrol treatments, the bacterial counts on the root surface were deceased on Day 1 and 3 after inoculation and in the root interior on Day 3 after inoculation compared with the control. The bacterial counts in the phytochemical treatments, either on the root surface or in the root interior, were significantly lower than that in the control group on Day 5 after inculcation. The inhibitory effects were visually shown in SMSA-agar plate (Figure 8).These results showed that resveratrol and coumarin had similar effects on the adhesion and colonization ability of *R. solanacearum* in the rhizosphere of tobacco plants. Both phytochemicals inhibited *R. solanacearum* colonization on the root surface and in the root interior. 

### 2.6. Control Efficacy of Phytochemicals to Bacterial Wilt of Tobacco

**Figure 9:**
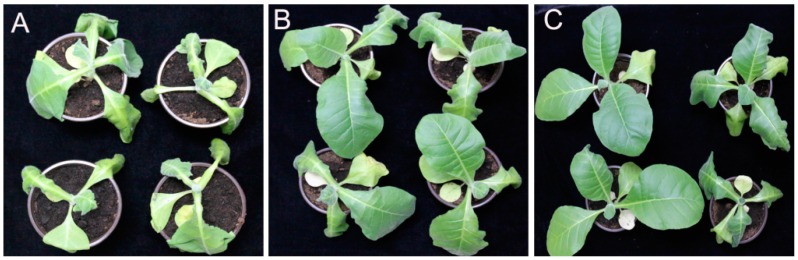
Control efficiency of sterile water (control) (**A**); resveratrol (**B**); and coumarin (**C**) in reducing wilting disease severity caused by *R. solanacearum* on tobacco seedlings at nine days post inoculation. The treatment concentration was 128 μg/mL. The experiments were performed a minimum of five times.

We performed a laboratory pot experiment to assess the antibacterial activity of phytochemicals against *R. solanacearum* in vivo on seven-week-old tobacco seedlings. The high concentration was selected for evaluating the control efficacy test of phytochemicals on bacterial wilt of tobacco. The results were recorded from the emergence of disease symptoms and expressed as disease incidence, disease index, and control efficacy. As shown in Table 4, the coumarin and resveratrol treatments suppressed the development of bacterial wilt, showing significant differences in disease incidence and disease index. The control efficiency of coumarin and resveratrol on tobacco bacterial wilts are 99%, 68%, and 37% and 100%, 85%, and 20% at 9, 13, and 19 days after root irrigation, respectively. After nine days post inoculation, the representative pictures of efficiently controlling the bacterial wilt disease are shown in Figure 9. On Day 13 after inoculation, the disease incidence and disease index in the control plants were 67% and 47%, respectively, both of which were markedly greater than those in the coumarin-treated (28% and 15%) and resveratrol-treated (22% and 7%) plants. Furthermore, the corresponding control efficacy of coumarin and resveratrol were 68% and 85%, respectively. When the disease incidence reached 100% in the control, the control efficacy was most significant in the phytochemical treatments (37% and 20%). Additionally, the emergence of wilting symptoms was markedly delayed, and the course of disease development was slower in phytochemical-treated plants compared with the control plants. Though the control efficacy was decreased with the deterioration of the disease after 19 days incubation, the values were still maintained at 37% and 20%, respectively. It is noteworthy that, when implementing pot experiment, higher inoculation concentration was needed in order to shorten experimental period, while stillensuring the occurrence of tomato bacterial wilt. The control efficacy in field application may be sufficiently higher than laboratory test. Therefore, based on the non-injured root inoculation, both phytochemicals significantly inhibit the incidence of tobacco bacterial wilt disease, demonstrating positive control efficacy by delaying or slowing the occurrence of disease.

## 3. Discussion

Coumarin and resveratrol, a wide group of naturally occurring compounds isolated from a variety of plants, due to their wide range of biological properties, including anticancer, anti-leukemic, antifungal and antibacterial activity, have attracted considerable researcher attention [25,37,38]. However, there have been few studies focused on the antibacterial activity of phytopathgen and its innovative application in the agriculture field. In this study, we aimed to investigate the antibacterial activity of two phytochemicals against *R. solanacearum* and their control efficacy towards tobacco bacterial wilt, which is one of the catastrophic soil-borne diseases around the world. In vitro evidence from the platecount method, followed by the measurements of MIC and MBC, manifested that resveratrol and coumarin exhibited high bactericidal activity against *R. solanacearum* in both liquid and solid culture. To some extent, these results are consistent with previous studies on antibacterial activity of the essential oils. Pare et al. reported that thymol, palmarosa oil, and lemongrass oil have markedly antibacterial effects and there was no bacteria detected in ginger plants in pot experiments, significantly reducing the incidence of bacterial wilt. However, no single treatment sufficiently controlled the disease after 180 days of growth [16]. Meanwhile, there were significant differences in the effects of the different phytochemicals and the growth inhibitory effects of phytochemical on *R. solanacearum* in NB medium are concentration dependent. A comparison of *R. solanacearum* growth in different treatments revealed that both resveratrol and coumarin can affect the normal growth cycle and inhibit the growth of *R. solanacearum*. A high concentration (128 μg/mL) of coumarin resulted strongest inhibitory effect with nearly no bacterial growth while different concentrations of resveratrol all showed inhibitory effects on bacterial growth.

Previous studies have shown that the cell membrane is the primary target for the antimicrobial activity of most phytochemicals [18,29,39]. These compounds interact with the lipid bilayer of the cytoplasmic membrane and increase the space between fatty acid chains, destabilizing the membrane structure and enhancing its fluidity and permeability [29,40]. Coumarin has been considered as a naturally active component of essential oil. Another study illustrated that the active components of the essential oil might interact with the cytoplasmic membrane, penetrating to the membrane-bound enzymes on the surface and the phospholipid bilayer, then disturb the subsequent macromolecules synthesis process, such as DNA, RNA, protein, or polysaccharides, eventually causing the death of the cells [41]. Presently, resveratrol and coumarin have shown to be active mainly against medical pathogens, especially methicillin-resistant *Staphylococcus aureus* (MRSA) and food-derived pathogens [39,40]. Thus far, relevant research on plant pathogens are still is rare, and no study has reported their antibacterial activity against *R. solanacearum* or the associated mechanism. In the current study, we observed the cell morphology of *R. solanacearum* by TEM images after treatment with two phytochemicals. The biocontrol mechanism is most likely due to the action of cell morphology deformation and cell membrane damage induced by the phytochemical. We found that in the presence of resveratrol, bacterial cells showed anon-uniform size and irregular shape, the cell surface was wrinkled, and some cells were ruptured. We speculate that the antibacterial action of two phytochemicals is also reflected in physical damage and impacts on cell metabolism. Paulo et al. reported the antibacterial effects of resveratrol against several dozen human pathogenic bacteria [40].They found that resveratrol can alter the morphological structure and DNA content and also affect the metabolic cycle of bacteria, which was in agreement with the antibacterial action of resveratrol and coumarin in our study. Similarly, Ultee et al. [42] indicated that carvacrol can interfere with H^+^ and K^+^ transport across the cell membrane of the foodborne pathogen *Bacillus cereus*, leading to ion gradient disorders and affecting the basic cellular functions, ultimately leading to cell death. La Storia et al. performed anatomic force microscopy analysis and proved that the cell membrane is the primary target site of carvacrol [43].The reduction in cell size, length and diameter adds to the evidence that carvacrol alters the cell surface structure of pathogenic bacteria. In short, TEM images illustrate that resveratrol and coumarin caused significant damage to *R. solanacearum* cells, showing incomplete cell morphology and a rough outer membrane. That may be because the polarity of the phenolic compounds affects the interaction between resveratrol and membranes [44]. These observations indicate that phytochemicals may inhibit the bacterial cell wall or destroy the outer membrane to change its permeability, leading to the leakage of intracellular substances and cell rupture or retraction (Figure 4). Like other polyphenols, they can also bind to membrane, disturbing the hydrophobic part of the lipid bilayer [44].Previous study also found sorbic acids can most likely interact with the headgroups and shallowly embed near the top of the phospholipid head groups [45]. Thus, the cells show an incomplete morphology and therefore decreased bacterial growth. These findings are of great significance to the study of new agricultural phytochemicals and the development of new pesticides.

Another explanation for the mode of action of resveratrol and coumarin may involve the prevention of biofilm and inhibition of bacterial motility (Figure 5 and Figure 6). It is well known that the *R. solanacearum*–host pathogenic interaction is generally described by a pathogenesis progress of strains of *R. solanacearum* into hosts generally involving three stages: moves to the host plant, attaches to the plant roots, and infects the cortex and colonizes the xylem parenchyma [46,47]. This invasion process results from the coordination of multiple pathogenic factors, including extracellular polysaccharides, a type III secretion system and its effectors, extracellular proteins, lipopolysaccharides, and a type IV flagellar system [48,49,50]. When pathogenic bacteria *R. solanacearum* colonized biotic or abiotic surfaces, surface motility and biofilm play essential role in plant root infection and early colonization process [49,51].

From our results, coumarin and resveratrol can inhibit the swarming motility of *R. Solanacearum* cells, even in the shorter incubation time of 24 h. Swarming motility, aflagella-dependent surface motility, is migratory of bacterial cells from inoculation point as a group on the surface of solid medium [52]. Once the bacteria motion are restrained, a series of behaviors will be affected including replication and recruitment that prompt the formation of aggregates of bacteria, then inhibit mature biofilm development [53], as observed in this study (Figure 6). Bacterial biofilms, as film-like composites, are a structured community of bacterial cells enclosed in a self-produced polymeric matrix and adherent to host surface, which consist of bacterial cells, extracellular polysaccharides, extracellular proteins, and water [33]. For plant pathogens, biofilm formation mainly plays a role in clogging the xylem conduit, resisting antimicrobial agents, and increasing colonization within a particular environment [25,54]. In the current study, we used a fluorescently labeled lectin (FITC-ConA) to enable the specific staining and qualitative analysis of polysaccharides in bacterial biofilm. We found that both coumarin and resveratrol inhibited biofilm formation by *R. solanacearum*, which was similar with some natural plant extracts such as cinnamon, protocatechualdehyde and eucalyptus essential oils [21,55]. Therefore, not surprisingly, *R. solanacearum* could not aggressively adhere to and colonizes tobacco roots surfaces, as shown in Table 3. We can speculate that the important antibacterial mechanism of resveratrol and coumarin is very likely through decreased levels of expression of a number of virulence factors related to cell motility and pathogenicity, suggesting decreased pathogenicity of the bacterium after treatment.

Further, laboratory pot experiments in vivo showed that both resveratrol and coumarin can suppress the adhesion ability of *R. solanacearum* cells and thereby delay or slow the occurrence of bacterial wilt of tobacco, the control efficacy of which were up to 68% and 85% at 13 days after inoculation, respectively. It seems that resveratrol and coumarin exhibited high efficacy for controlling tobacco bacterial wilt before the bacteria blocked the plant vascular. This can be expected as the phytochemicals might thereby perform as a potential natural antimicrobial agent in bacterial wilt management. Though the values were reduced after 19 days of irrigating, continuous irrigation may be resorted to manage the bacterial wilt. These results may be related to the complex pathogenic regulatory system of *R. solanacearum*. The process of bacterial wilt is controlled by a complex pathogenic regulatory system via the coordinated expression of multiple genes. Extensive trials are needed to further explore the effect of phytochemicals on the pathogenic genes of *R. solanacearum.*

## 4. Materials and Methods

### 4.1. Bacterial Strain and Culture Conditions

The test *R. solanacearum* strain (Biovar3, Phylotype I) was obtained from Laboratory of Natural Product Pesticide of Southwest university, which was identified to be a highly pathogenic strain. The strain was cultured on NA agar medium (yeast extract 1.0 g, beef extract 3.0 g, peptone 5.0 g, glucose 10.0 g, agar 20.0 g, deionized water 1000 mL, pH 7.0) at 30 °C for 48 h. Single colonies were picked and transferred into NB liquid medium (NA medium without agar) for 12–16 h. The cells were harvested, by centrifugation, at an optical density at 600 nm (OD_600_) of 1.0 and stored at −80 °C before use.

Resveratrol and coumarin used in this study were purchased from Shanghai Yuanye Bio-technology (Shanghai, China) and had ≥98% purity (by HPLC analysis). Stock solutions (20 mg/mL resveratrol or coumarin) were prepared in dimethyl sulfoxide (DMSO, Sigma-Aldrich, St. Louis, MO, USA) and filter-sterilized through a bacterial filter before use.

### 4.2. Minimum Inhibitory Concentration (MIC) and Minimum Bactericidal Concentration (MBC) Assays

The bacteriostatic and bactericidal activities of resveratrol and coumarin against *R. solanacearum* were examined referring to the typical microdilution method (ISO 10932|IDF 223:2010) to determine the minimal inhibitory concentration (MIC) and minimal bactericidal concentration (MBC). The stock solution of resveratrol or coumarin (20 mg/mL) was added to 10 mL of NB liquid medium to produce phytochemical-containing media with final concentrations of 1, 2, 4, 8, 16, 32, 64, and 128 μg/mL. The phytochemical-containing media were inoculated with 10 μL of bacterial suspension at OD_600_ = 1.0 (≈10^9^ cfu/mL) and incubated at 30 °C with shaking at 180 r/min for 24 h. The OD values of the culture solution were read using a Thermo electron Nicolet Evolution 300 UV-Vis spectrophotometer (Thermo Sci. Inc., Wilmington, NC, USA). The minimum inhibitory concentration (MIC) was defined as the lowest concentration of phytochemicals that inhibited bacterial growth.

The minimum bactericidal or lethal concentration (MBC) was determined based on the viability of the strain on solid medium. NA medium was melted and cooled at room temperature to 45 °C. Resveratrol or coumarin was then added to obtain phytochemical-containing media at final concentrations of 10–250 μg/mL. The phytochemical-containing media were thoroughly mixed before being poured into 9-cm diameter Petri dishes (15 mL each). A bacterial suspension at OD_600_ = 1.0 (≈10^9^ cfu/mL) was then diluted to 1 × 10^5^ cfu/mL, and 100 μL of diluted suspension was spread evenly on the phytochemical-containing plates. The inoculated plates were inverted and incubated at 30 °C for 96 h. The experimental results were then photographed and recorded. The MBC was defined as the lowest concentration of phytochemical that yielded no colony growth. DMSO with a final concentration of 0.1% was used as the negative control, and a blank control (Control) was also prepared in each experiment.

### 4.3. Growth Curve Assay

The stock solution of resveratrol and coumarin were pipetted into 100 mL of NB liquid medium to produce phytochemical-containing media with final concentrations of 128, 64, 32, 16, and 8 μg/mL, respectively. Phytochemical-containing medium was inoculated with 100 µL of bacterial suspension and incubated at 30 °C with shaking at 180 r/min for 36 h. The OD_600_ values of the samples were measured every 2 h during incubation using a Nicolet Evolution 300 UV-Vis spectrophotometer. A solvent control was prepared with DMSO at a final concentration of 0.1%, and a blank control was also included in each experiment. The OD_600_ values (vertical axis) were plotted against the sampling time (horizontal axis) to generate growth curves for *R. solanacearum* under the effect of the phytochemicals. The experiments were repeated multiple times, and the results were expressed as arithmetic means.

### 4.4. Observation of Bacterial Cell Morphology by TEM

To determine the antibacterial efficacy of the both phytochemicals and the morphological changes of the bacteria, TEM observation was performed on the tested bacteria according to the method as described previously [56]. First, the stock solution of resveratrol and coumarin (64 and 32 μL; 20 mg/mL each) were added into 10 mL of NB medium to obtain phytochemical-containing media with final concentrations of 128 μg/mL, respectively. Then, 10 μL of bacterial suspension (OD_600_ = 1.0) was inoculated and thoroughly mixed. The culture mixture was incubated at 30 °C with shaking at 180 r/min for 2 h. Twenty-four hours later, the culture mixture was dispensed into 1.5-mL centrifuge tubes and centrifuged at 6000 r/min for 5 min to collect the cells. The cell pellet was resuspended in 0.1 M phosphate-buffered saline (PBS, pH 7.2), washed three times with PBS to remove the impurities, and the cell concentration was adjusted to 10^7^–10^8^ cfu/mL finally. Bacterial cells were resuspended in 1 mL of 2.5% glutaraldehyde, and the cells were fixed overnight at 4 °C. After that, the cells were collected by centrifugation at 6000 r/min for 5 min and dehydrated using an ethanol a serie of gradient (1 mL; 30%, 50%, 70%, 85%, and 100%). One drop of the cell suspension was evenly dispersed on foil, freeze-dried, and coated with gold. Thin sections were placed on the copper grids and Cell morphology was observed and photographed under Tecnai G20 microscopy (FEI, Brno, Czech Republic).

### 4.5. Bacterial Motility Assay

Motility assays were undertaken in petri dishes (polystyrene, diameter of 90 mm), referred to the previous report [53]. The stock solution of resveratrol or coumarin (20 mg/mL) was added to the semi-solid medium (0.35% agar) (SMM) at 45 °C to obtain final concentrations of 64, 32, 16, and 8 μg/mL. Fifteen milliliters of phytochemical-containing medium was dispensed into each Petri dish. Five microliters of bacterial suspension at OD_600_ = 0.1 (≈3 × 10^8^ cfu/mL) was dropped onto the prepared culture plates and spread evenly. The plates were kept horizontal during incubation to prevent experimental errors caused by the flow of the semi-solid medium. The diameters of the swarming motility zones around the bacterial colonies were measured after incubation (30 °C) for 24 and 48 h, respectively. Bacterial motility was calculated as follows:

Swarming motility diameters = Measured diameter (mm) − Initial inoculation diameter (6 mm)
(1)

A solvent control was prepared with DMSO at a final concentration of 0.1%, and a blank control was also included in each experiment. The experiment was repeated three times.

### 4.6. Bacterial Biofilm Formation Assay

The bacterial culture under different treatment was dispensed into sterile polystyrene microtiter plate (96-well plates) and mixed. No culture was added to wells at the edge of the plates. All experiments were performed in triplicate at least. All the 96-well plates were incubated at 30 °C under 24-h static culture. After that, the culture was removed carefully and gently with eppendof and, immediately, the 96-well plates were washed with 200 μL of distilled water. Bacterial biofilm was stained with 220 μL of 0.1% crystal violet and incubating at room temperature for 30 min without shaking. Then, crystal violet was removed, and the biofilm was washed twice with 200 μL of distilled water. After the floating color was eliminated, the biofilm was dried for 30 min at room temperature. Then, 200 μL of 95% ethanol was added to dissolve and adsorb the crystal violet. The absorbance of the solution at 490 nm was measured using a microplate reader to reflect biofilm formation by *R. solanacearum* under the experimental conditions [57]. The same procedure was followed to measure biofilm formation at 48 and 72 h. Tests were performed in triplicate.

### 4.7. Biofilm Observations by Fluorescence Microscopy

We further observed *R. solanacearum* biofilm formation by staining the main extracellular component of the biofilm using fluorescein isothiocyanate-conjugated concanavalin A (FITC-ConA), which can emit green fluorescence and be imaged via fluorescence microscopy [58]. Firstly, phytochemical-containing media with final concentrations of 64 and 128 μg/mL were prepared. Phytochemical-containing medium was then inoculated with 10 μL of bacterial suspension (OD_600_ = 1.0) and thoroughly mixed. Next, a 10 mm × 10 mm sterile coverslip was placed into each well of the 24-well plates as a carrier for biofilm adhesion, followed by the addition of 1 mL of culture mixture per well. After the inoculated plates were covered with plastic wrap and incubated for 12 h at 30 °C, the coverslips were removed and washed with PBS and then fixed with 2.5% glutaraldehyde for 4 h at 4 °C. After removed the residual fixative solution, the coverslips were stained with FITC-ConA in the dark for 30 min at 4 °C, followed by three washes with PBS to remove the redundant dye. Then, the coverslips were dried on absorbent paper and mounted on glass slides using 40% glycerol. Biofilms were observed on an inverted fluorescence microscope (Eclipse Ti, Nikon, Tokyo, Japan).

### 4.8. Bacterial Adhesion Test

The stock solutions of resveratrol and coumarin (20 mg/mL) were diluted to 128 μg/mL with sterile water. Then, 30 mL of diluted phytochemical solution were respectively evenly irrigated into tobacco roots. Twelve hours later, *R. solanacearum* cells were inoculated via non-injured root inoculation at OD_600_ = 0.1 (≈10^8^ cfu/mL), 10 mL per plant. The inoculated tobacco seedlings were cultivated in a plant growth chamber at a temperature of 30 ± 1 °C, relative humidity of 85%–90%, and light period of 14 h. Control plants were irrigated with sterile water in the experiment. Each treatment was designed nine plants and three replicates. One day after inoculation, three plants, which were randomly selected from each treatment, were washed to obtain clean and intact roots. The root weights were recorded. The weighed tobacco roots were placed in a flask containing 50 mL of sterile water and incubated for 30 min at 30 °C, with shaking at 180 r/m. The roots were then rinsed with sterile water and thoroughly mixed with sterile water in the flask. The mixed solution was allowed to stand for 5 min, and 1 mL of tobacco root exterior bacterial suspension was taken from the mid-upper portion with a pipette.

The tobacco roots were surface-dried with absorbent paper and disinfected for 20 min in a Petri dish containing 75% ethanol. After that, the disinfected roots were thoroughly ground in a sterile mortar. Ten milliliters of sterile water was added to the mortar to thoroughly mix the root homogenate. The mixture was allowed to stand for 5 min, and 1 mL of supernatant was then taken as the tobacco root interior bacterial suspension. The bacterial suspensions obtained from the tobacco roots were prepared in serial dilutions at 10^−1^ to 10^−7^. The number of *R. solanacearum* cells in the suspensions was measured by plate counting on selective SMSA medium. Three dilutions of the root exterior (10^−2^ to 10^−4^) and interior bacterial suspensions (10^−1^ to 10^−3^) were evenly spread on duplicate SMSA plates (0.1 mL each). The inoculated plates were incubated for 48 h at 30 °C and *R. solanacearum* colonies were recorded. The number of *R. solanacearum* cells in the bacterial suspensions was calculated at different dilutions to obtain root-exterior and interior bacterial counts. The same procedure was repeated at 3 and 5 days after inoculation. However, the dilutions were changed to 10^−3^ to 10^−5^ for root-exterior bacterial suspension and 10^−2^ to10^−4^ for root-interior bacterial suspension on Day 3 and 10^−3^ to 10^−5^ for both root exterior and interior bacterial suspensions on Day 5.

### 4.9. Controlling Tobacco Bacterial Wilt In Vivo under Greenhouse Conditions

Firstly, the uniformly grown four-week-old tobacco seedlings were selected to carry out the effects of resveratrol and coumarin on tobacco bacterial wilt. Furthermore, 30 mL of diluted phytochemical solution (128 μg/mL) were respectively evenly irrigated into tobacco root soil. Deionized water without phytochemicals or bacteria was set as blank control in this experiment. Nine tobacco seedlings were carried out for each treatment. Twelve hours later, *R. solanacearum* was inoculated via non-injured root inoculation at OD_600_ = 0.1 (≈10^8^ cfu/mL) per plant. The inoculated tobacco seedlings were cultivated in a plant growth chamber at a temperature of 30 ± 1 °C, relative humidity of 85%–90%, and light period of 14 h. Following the inoculation of *R. solanacearum*, the disease occurrence of the tobacco seedlings and disease index were investigated at 7, 9, 13 and 19 days since the first observation of bacterial wilt diseased plants (7 days in our experiment). Bacterial wilt disease classification of tobacco plants in the laboratory was evaluated and recorded according to the “Protocols of Disease Investigation and Classification” [59]. The disease incidence, disease index, and control efficacy were calculated using Equations (2)–(4) as follows:

Disease incidence (%) = Number of diseased plants/total number of plants × 100
(2)

Disease index = [Σ (Number of diseased plants × number of diseased plants)/(total number of plants × representative value of the highest grade)] × 100
(3)

Control efficacy (%) = (Disease index of the control − disease index of the treatment)/disease index of the control × 100
(4)

### 4.10. Statistic Analysis

All the experiments were performed in triplicate and the results were expressed as mean values ± SD (standard deviation). Statistical analysis was implemented using Statistical Product and Service Solutions software (SPSS 11.0) (SPSS Inc., Chicago, IL, USA). The differences between the groups were assessed using the analysis of variance test. The results were considered statistically significant when the *p* value was <0.05 or <0.01. 

## 5. Conclusions

In summary, two phytochemicals, resveratrol and coumarin, significantly inhibited the propagation of *R. solanacearum* and demonstrated a bactericidal effect in vitro and in vivo. On the one hand, the cell membrane structure of *R. solanacearum* was damaged after phytochemical treatment, thereby affecting bacterial growth. On the other hand, both resveratrol and coumarin inhibited the formation of bacterial biofilms and impeded the motility of *R. solanacearum*, which plays an important role in preventing *R. solanacearum* infection of tobacco plants. Furthermore, in vitro experiments demonstrated that resveratrol and coumarin inhibited early adhesion and colonization by *R. solanacearum* during infection, thereby significantly controlling bacterial wilt of tobacco. However, this study was limited to pot experiments, and it is therefore necessary to perform field trials on the prevention and control of bacterial wilt. Meanwhile, in-depth studies are needed to further elucidate the antibacterial mechanism of resveratrol and coumarin.

## Figures and Tables

**Table 1:** Several phytochemicals with inhibitory activity to *R. solanacearum.*

Family	Plant Species	Active Ingredients
Poaceae	*Phyllostachys pubescens Mazel*	Bamboo tar
	*Cymbopogon martinii*	Palmarosa Oil
Lamiaceae	*Thymus serpyllum L.*	Essential oil
	*Origanum vulgare “Rogeukuppel”*	
	*Saluia plebeia R. Br.*	Ethanol extract
	*Leucas aspera (Willd.) Link*	
Apocynaceae	*Herba Catharanthi rosei*	Ethanol extract
	*Hunteria zeylanica*	
Rutaceae	*Atalantia buxifolia*	Ethanol extract
	*Micromelum falcatum*	
	*Clausena lansium (Lour.) Skeels*	Seed extracts
Liliaceae	*Allium sativum L.*	Root exudates
Asteraceae	*Amaranthus tricolor L.*	Ethanol extract
	*Eupatorium adenophorum Spreng*	Leaf exudates
	*Elephantopus tomentosus L*	Ethanol extract
Magnoliaceae	*Magnolia officinalis Rehd. et Wils.*	Phenolic compounds
Others	*Stemona sessilifolia (Miq.) Miq*	Petrol ether extract
	*Casuarinaequisetifolia L*	Ethanol extract
	*Sophoraflavescens*	Alkaloid
	*Lithospermum erythrorhizon*	Chloroform extract
	*Toxicodendron sylvestre*	Trimethylgallic acid
	*Litsea cubeba*	Ethanol extract

**Table 2:** Effects of different compounds on the growth of *R. solanacearum* after 24 h incubation.

Compounds	Concentration (μg/mL)							
	128	64	32	16	8	4	2	1
resveratrol	+	+	+	+	+	+	+	-
coumarin	+	+	+	+	+	-	-	-

+ represented that compound has inhibitory effect on the growth of *R. solanacearum,* - represented that compound has no effect on the growth of *R. solanacearum.*

**Table 3:** Effects of coumarin and resveratrol on rhizosphere colonization of *R. solanacearum* in tobacco.

Treatments	On the Root Surface			Within the Root		
	One Day after Inoculation (cfu/g)	Three Days after Inoculation (cfu/g)	Five Days after Inoculation (cfu/g)	One Day after Inoculation (cfu/g)	Three Days after Inoculation (cfu/g)	Five Days after Inoculation (cfu/g)
Control	1.03 × 10^6^ b	6.26 × 10^6^ a	1.79 × 10^8^ a	0.00	1.50 × 10^5^ b	5.2 × 10^6^ a
Coumarin	9.30 × 10^5^ b	4.31 × 10^6^ a,b	1.83 × 10^7^ c	0.00	4.12 × 10^4^ c	2.44 × 10^6^ b
Resveratrol	3.44 × 10^5^ c	3.58 × 10^6^ b	2.64 × 10^7^ c	0.00	1.41 × 10^5^ b	2.41 × 10^6^ b

Values represent means of three independent replicates ± SD. Different letters (a, b, c) within a column indicate statistically significant differences between the means (*p* < 0.05).

**Table 4:** Inhibition effects of coumarin and resveratrol against tobacco bacterial wilt under greenhouse.

Days after Inoculation	Control			Coumarin Treatment			Resveratrol Treatment		
	Disease Incidence (%)	Disease Index	Control Efficacy (%)	Disease Incidence (%)	Disease Index	Control Efficacy (%)	Disease Incidence (%)	Disease Index	Control Efficacy (%)
7	0	0	-	0	0	100	0	0	100
9	13	3	-	0.3	0	99	0	0	100
13	67	47	-	28	15	68	22	7	85
19	100	92	-	80	58	37	86	74	20
